# Supplementary material for: The Integration of Metabolomics, Electronic Tongue, and Chromatic Difference Reveals the Correlations between the Critical Compounds and Flavor Characteristics of Two Grades of High-Quality Dianhong Congou Black Tea
Source: Metabolites. 2023 Jul 20;13(7):864. doi: 10.3390/metabo13070864 (PMC10385030; doi:10.3390/metabo13070864)
Supplement: Supplementary file 1 [file metabolites-13-00864-s001.zip › metabolites-2501543-supplementary.pdf]

Materials and Methods

Human sensory evaluation

First, the tea samples were numbered randomly, and 200 g of each tea sample was put into a white square plate for the dry tea appearance evaluation. Second, 4 g of each tea sample was put into a white clean porcelain cup and brewed with 150 mL boiled water. After 5 min, the tea infusion was filtered into a white clean porcelain bowl and the tea infused leaf was kept in the cup for the evaluation of liquor color, aroma, and taste. Third, the infused leaf was placed into a white porcelain plate for the evaluation of infused leaf appearance. Then, the comments and scores of five sensory factors (dry tea appearance, liquor color, aroma, taste, and infused leaf) of tea were given by five certificated tea assessors referring to the Tea Vocabulary for Sensory Evaluation (GB/T 14487-2017) and Methodology for Sensory Evaluation of Tea (GB/T 23776-2018). And then, the final score of each sensory factor was taken as the average of the scores given by the 5 reviewers. Finally, the overall score of each tea sample was calculated according to a 100-point scoring system: Total score (100%) = dry tea appearance (25%) + liquor color (10%) + aroma (25%) + taste (30%) + infused leaf appearance (10%).

Table S1. Detailed sensory comments and scores (dry tea appearance, liquor color, aroma, tea taste, infused leaf appearance, and total score) of the two grades of high-quality DCT evaluated by human sensory evaluation.

Group	No.	Dry tea appearance (25 %)		Liquor color (10 %)		Aroma (25 %)		Taste (30 %)		Infused leaf (10 %)		Total score (100%)
		comment	score	comment	score	comment	score	comment	score	comment	score	
BDCT	1	tight and heavy, black bloom, golden tippy	96	red, bright	90	sweet aroma	82	heavy brisk with umami	86	red, bright	86	87.9
	2	tight and heavy, black bloom, golden tippy	96	red, bright	91	high sweet aroma	88	heavy brisk with umami	87	red, bright	88	90
	3	tight and heavy, black bloom, golden tippy	96	red, bright	92	fresh sweet aroma	85	slightly brisk with umami	84	red, bright	90	88.65
	4	tight and heavy, black bloom, golden evenly tippy	97	slightly red, bright	86	slightly sweet aroma	80	heavy brisk with umami and fruity-like taste	84	red, bright	90	87.05
	5	tight and heavy, black bloom, golden evenly tippy	96	red and brilliant, bright	96	high sweet aroma with fresh	92	slightly brisk with umami and fruity-like taste	82	red, bright	90	90.2
	6	tight and heavy, black bloom, golden evenly tippy	96	slightly red, bright	88	sweet and flowery aroma	94	strong, fresh and brisk, fruity-like taste	90	red, bright	88	92.1

	7	fat and blod bud, golden evenly tippy	97	orange red, bright	91	sweet aroma	82	less mellow, brisk and umami	80	red, bright	86	86.45
	8	slightly tight and heavy, black bloom, golden tippy	96	slightly red, bright	88	fresh and sweet aroma	89	heavy brisk with umami and fruity like taste	85	red, bright	89	89.45
	9	tight and heavy, black bloom, golden evenly tippy	97	slightly red, bright	88	fresh sweet, flowery aroma	84	strong, umami, fruity-like taste, with slightly brisk,	83	red, bright	87	87.65
	1	tight and heavy, bent, black bloom, slightly golden tippy	94	red and brilliant, bright	94	sweet aroma	84	slightly thick, sweet-mellow	82	red, bright	92	87.7
	2	tight and heavy, bent, black bloom, slightly golden tippy	95	red, slightly bright	87	sweet aroma	86	thick and mellow, sweet	88	red, slightly bright	86	88.95
	3	tight and heavy, bent, black bloom, slightly golden tippy	94	red, slightly bright	87	sweet aroma	88	thick and mellow, sweet	87	red, slightly bright	86	88.9
	4	tight and heavy, bent, black bloom, slightly golden tippy	94	red, slightly bright	88	less sweet aroma	80	slightly sweet-mellow	82	red, bright	88	85.7
SDCT	5	tight and heavy, bent, black bloom, slightly golden tippy	94	red, slightly bright	88	sweet aroma	84	slightly thick and sweet-mellow	84	red, bright	87	87.2
	6	tight and heavy, bent, black bloom, with golden tippy	93	red, slightly bright	87	sweet aroma	84	sweet, thick and mellow	85	red, slightly bright	86	87.05
	7	tight and heavy, bent, black bloom, with golden tippy	93	red and brilliant	94	high sweet aroma	92	thick and mellow	88	red, bright	92	91.25
	8	tight and heavy, bent, black bloom, with golden tippy	93	red, slightly bright	88	sweet aroma	84	slightly sweet-mellow	82	red, slightly bright	85	86.15
	9	tight and heavy, bent, black bloom, slightly golden tippy	92	red, slightly bright	87	high sweet aroma	89	thick and sweet-mellow	87	red, bright	89	88.95

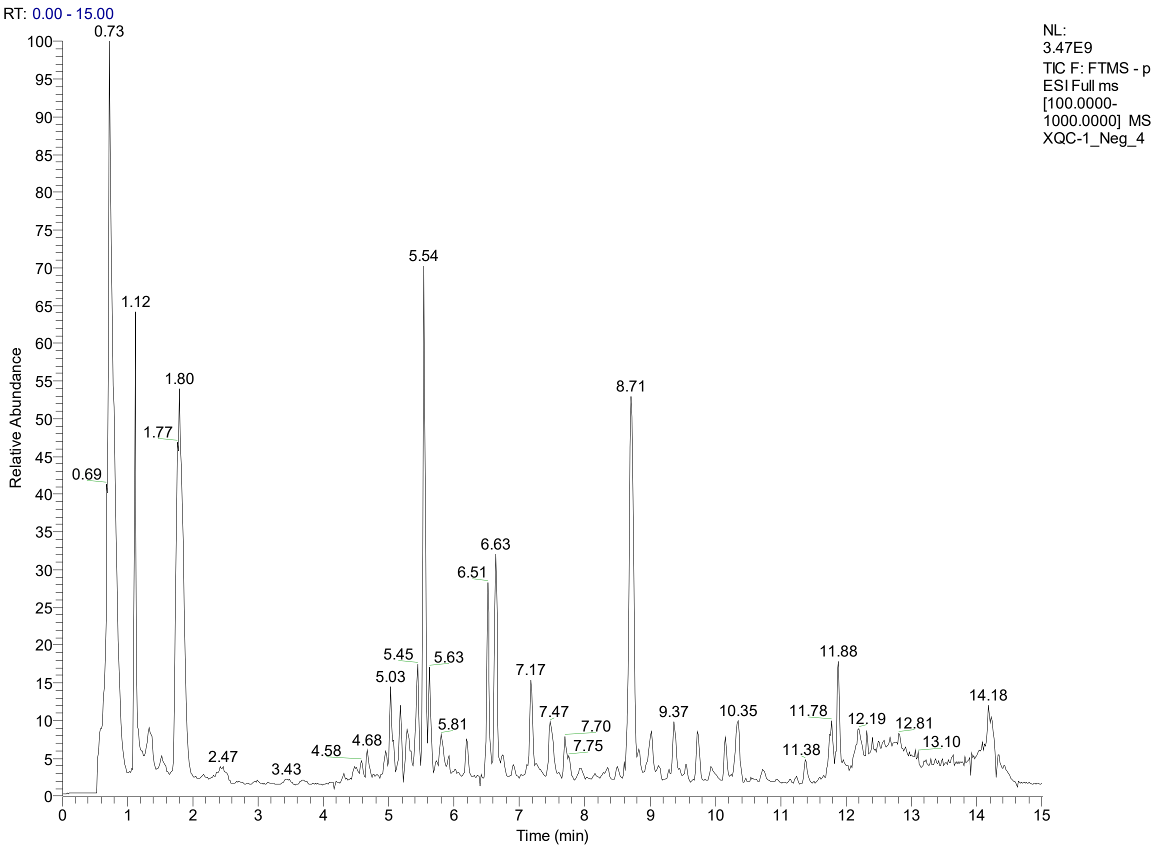

Figure S1. The typical chromatogram of total ions acquired from the metabolomics analysis.

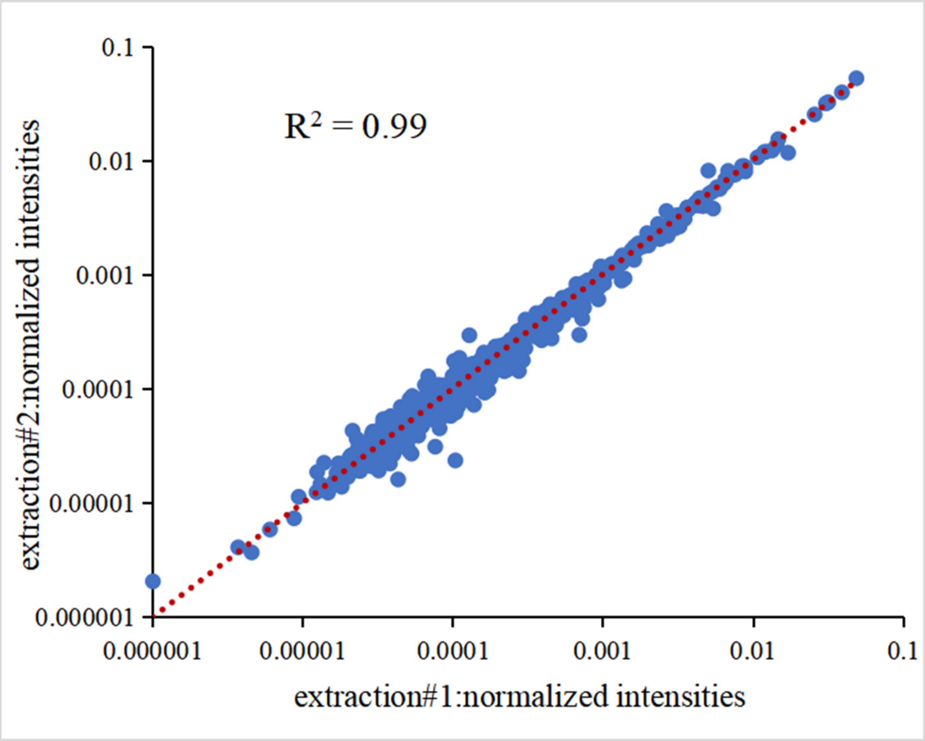

Figure S2. Scatter plot of normalized intensities of all detected ions in the two replicate extractions.
